# Cerebral venous sinus thrombosis as a complication of heparin-induced thrombocytopenia in myasthenia gravis: A rare and complex case

**DOI:** 10.5339/qmj.2025.59

**Published:** 2025-06-09

**Authors:** Majd A. AbuAlrob, Abdullah Mantawil Gumander, Khaled Zammar, Abdullah I AL Qazakzeh, Sadi Y. Alnakhala, Osama M. Khalil, Suhail Hussain

**Affiliations:** 1 Neuroscience Institute, Hamad Medical Corporation, Doha, Qatar; 2 Department of Medicine, Hamad General Hospital, Doha, Qatar *Email: majdaiabualrob72@gmail.com

**Keywords:** Cerebral venous sinus thrombosis, heparin included thrombocytopenia myasthenia gravis

## Abstract

**Background:**

Cerebral venous sinus thrombosis (CVST) is an uncommon yet critical complication, especially when arising from heparin-induced thrombocytopenia (HIT). In patients with preexisting conditions such as myasthenia gravis (MG), this correlation adds further complexity to clinical management and outcomes.

**Case presentation:**

We report a unique case of CVST induced by HIT in a patient with an established diagnosis of MG. Following plasma exchange therapy, which included heparin administration, the patient developed symptoms indicative of CVST. Diagnostic imaging confirmed thrombosis in the cerebral venous sinuses. Management involved the immediate discontinuation of heparin and the initiation of fondaparinux, leading to effective anticoagulation and clinical improvement.

**Discussion:**

This case illustrates the rare intersection of CVST and HIT within the context of MG, underscoring the potential risks associated with heparin therapy in vulnerable patient populations. Early recognition of the signs is essential, as these conditions, in combination, demand prompt and specialized interventions to prevent serious complications.

**Conclusion:**

The successful management of this complex case demonstrates the importance of heightened awareness and proactive strategies in patients with MG undergoing heparin therapy. This report advocates for careful monitoring and tailored treatment to mitigate risks in similarly complex clinical scenarios.

## Introduction

Cerebral venous sinus thrombosis (CVST), though rare, is a serious cerebrovascular disorder that can potentially be life-threatening, with an annual incidence of approximately 3–4 cases per million in the general population. Despite its relative infrequency, CVST accounts for 0.5%–1% of all strokes. This condition predominantly affects a younger demographic, particularly females, due to hormonal influences, pregnancy, and the use of oral contraceptives.^1^ The pathophysiology of CVST involves thrombus formation within the dural venous sinuses, leading to venous congestion, hemorrhagic infarctions, and elevated intracranial pressure.^2^

Advancements in neuroimaging have presented diagnostic challenges, as CVST manifests with highly variable clinical presentations. Persistent headaches are among the most common symptoms, resembling tension-type and migraine headaches, and may persist from days to weeks.^1^ In severe cases, venous infarction or hemorrhage can result in neurological deficits such as hemiparesis, aphasia, or seizures.^3^ Papilledema, often linked to elevated intracranial pressure, may manifest as mental status changes, ranging from confusion to coma, in severe CVST cases.^4^ Given the nonspecific nature of these symptoms, early diagnosis often relies on advanced imaging techniques, specifically magnetic resonance imaging with venography.^1^ Timely intervention with anticoagulation therapy can markedly improve patient outcomes.^5^

Heparin-induced thrombocytopenia (HIT), by contrast, is an uncommon but serious complication of heparin therapy, occurring in 1%–3% of patients receiving unfractionated heparin and 0.1%–1% of those treated with low-molecular-weight heparin.^6^ HIT is characterized by a reduction in platelet counts by 30%–50% within 5–14 days of heparin administration.^7^ Although it involves thrombocytopenia, HIT paradoxically promotes thrombotic events, predominantly venous and, to a lesser extent, arterial thrombosis.^8^

Recent studies suggest a possible link between myasthenia gravis (MG) and HIT, where the autoimmune and neuromuscular dysfunctions associated with MG may be exacerbated by immune complexes related to HIT.^9^ This hypercoagulable state could potentially worsen MG symptoms and elevate overall morbidity.^9^ In this report, we present a case of a female patient with MG who developed CVST as a rare complication of HIT following plasma exchange therapy.

Informed consent was obtained from the patient for the publication of this case report and associated images. The study was approved by the Medical Research Center at Hamad Medical Corporation, under reference number MRC-04-24-551.

## Case Presentation

A 58-year-old woman with a 12-year history of MG, previously managed with thymectomy and ongoing oral pyridostigmine, presented to the emergency department of a public hospital in Qatar. She had recently completed five sessions of plasmapheresis in Iran, where heparin was administered to prevent extracorporeal clotting, though the exact dose was unknown. She reported no prior use of heparin or heparin-containing products.

Four days after her last plasmapheresis session, the patient arrived at the emergency department with a sudden onset of headache, accompanied by weakness and numbness on the left side of her body. Physical examination showed that her mental status was intact, with objectively confirmed left-sided weakness.

**Figure 1:**
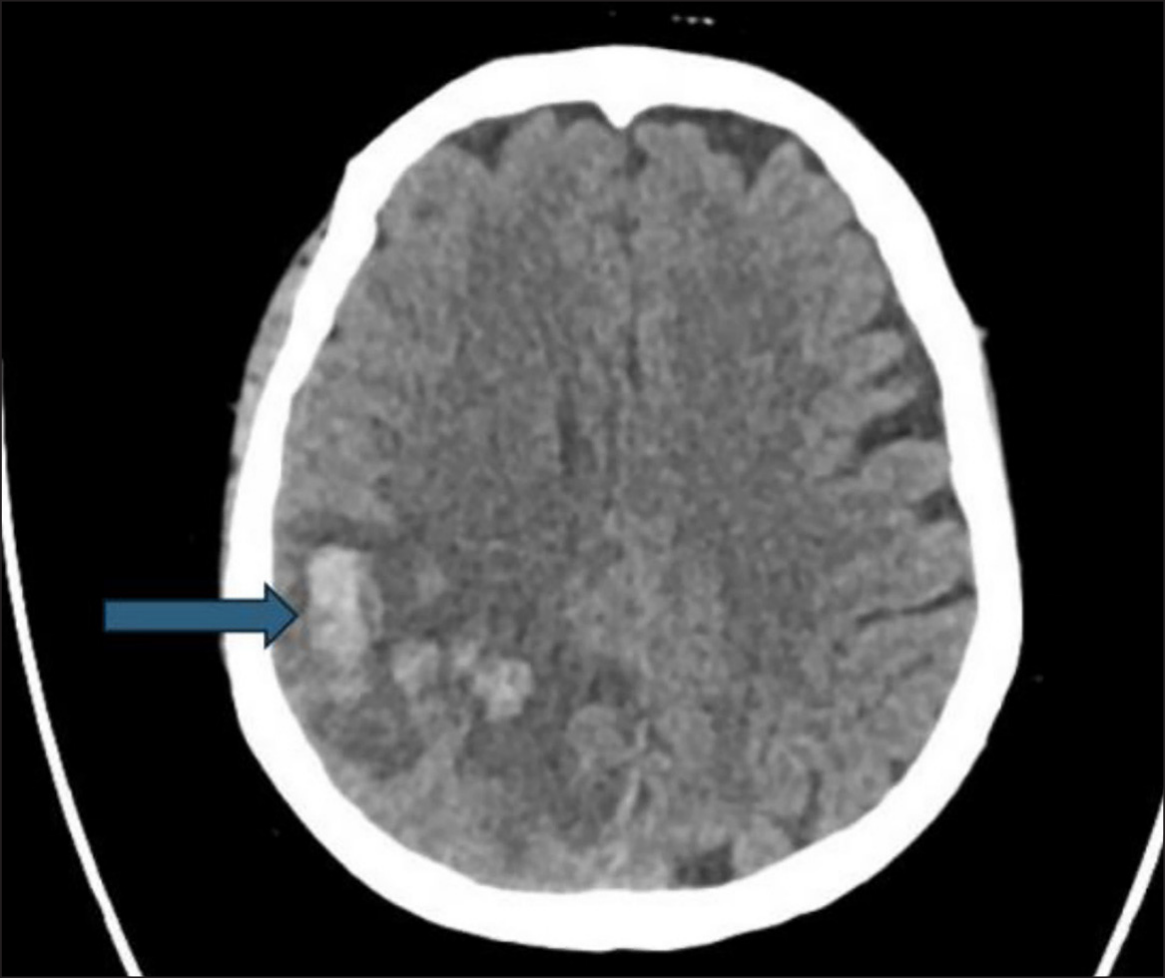
CT of the head showing multiple hyperdense lesions in the right parietal regions, consistent with hemorrhagic infarcts.

Imaging studies, including a computed tomography (CT) scan of the head, CT venogram, and CT angiogram, revealed multiple hyperdense lesions in the right parietal lobe, consistent with intraparenchymal hemorrhages, associated with edema and a 4-cm midline shift (Figure 1).

**Figure 2:**
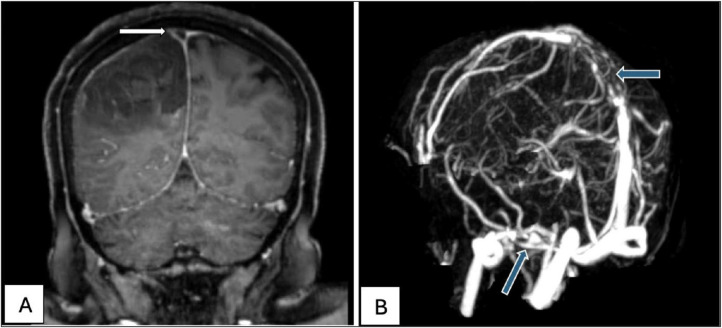
(A) A coronal cut magnetic resonance venography (MRV), showing a filling defect in the posterior portion of the superior sagittal sinus. (B) MRV of the brain posterolateral view demonstrating a 7-cm filling defect in the superior sagittal sinus (horizontal arrow). There is also evidence of a filling defect in the right transverse sinus (oblique arrow).

Additionally, the CT venogram showed a filling defect in the posterior part of the superior sagittal sinus extending to the left transverse sinus, indicative of cerebral venous thrombosis. MRV further confirmed the presence of cerebral sinus thrombosis, showing a 7-cm filling defect in the superior sagittal sinus and an additional defect in the right transverse sinus (Figures 2A and B). Laboratory investigations revealed thrombocytopenia, with a platelet count of 99 × 10³/μL, anemia (hemoglobin of 10.2 g/dL), and reticulocytosis (reticulocyte percentage of 3.3%). Given her recent heparin exposure and a high probability of HIT (estimated at ~65% based on the 4Ts score), the patient was started on fondaparinux 7.5 mg daily to normalize her platelet count, prevent further thrombotic events, and alleviate symptoms. On day two following admission, HIT antibody testing confirmed the presence of HIT antibodies, with a level of 4.7 U/mL.

Three days after initiating fondaparinux, her platelet count improved to 315 × 10³/μL. She continued to receive subcutaneous fondaparinux for 4 days, with progressive improvement in her headache and residual weakness. Five days following her presentation, the patient was discharged in stable condition on oral apixaban, prescribed for a total duration of 6 months, with plans for outpatient follow-up to monitor her recovery and manage anticoagulation therapy.

## Discussion

This case report highlights the rare yet serious prothrombotic complication of HIT manifesting as CVST, a combination that is scarcely documented in the existing literature.^10^ The incidence of thrombotic events in HIT has an estimated frequency of up to 50% in these patients.^6,11^ HIT occurs by the production of IgG antibodies to platelet factor 4 bound to heparin and leads to an activation of platelets, thrombin generation, and paradoxical thrombosis despite thrombocytopenia.

The rarity of HIT presenting with CVST makes this case particularly significant. The usual manifestations of HIT are deep vein thrombosis and pulmonary embolism, but CVST is an uncommon and severe manifestation; it occurs in very few cases. Warkentin^11^ stated that the relative frequency of isolated CVST complicating HIT is rare. The exact mechanisms that lead to such bizarre thrombotic events in HIT remain poorly understood, but it is felt that the highly prothrombotic state induced by HIT could affect any venous or arterial bed.^12^

There is high suspicion of HIT in this patient, given the thrombocytopenia and a high 4Ts score, which portends the likelihood of HIT with reasonable sensitivity. A further positive test for HIT antibody supports the finding of this condition. HIT presents therapeutic challenges because continued heparin use can worsen thrombosis. In this case, the coexistence of CVST was a rare manifestation of HIT, which further complicated the management approach.

In this situation, HIT management entails immediate discontinuation of heparin and commencement of a nonheparin anticoagulant. Fondaparinux, a synthetic pentasaccharide that does not cross-react with HIT antibodies, was chosen in this case for its effectiveness in preventing further thrombotic events. Studies confirm its role as a suitable alternative, reducing the risk of recurrence without heparin-related complications. The patient’s rapid platelet recovery and neurological stabilization validate this approach. Long-term anticoagulation with direct oral anticoagulants (DOACs), such as apixaban, has recently been shown to be a feasible option for HIT management, given their ease of management and safety profile in long-term anticoagulation when compared to warfarin, especially for complex thrombotic presentations like CVST.^13,14^ The decision to continue anticoagulation for 6 months was based on guidelines considering an extended course of anticoagulation for patients with provoked CVST and who have persistent risk factors according to Ferro et al.^1^

## Conclusion

This case demonstrated a rare instance of CVST caused by HIT, successfully managed with fondaparinux and long-term apixaban, resulting in significant recovery.

Overall, managing a patient with both MG and HIT presents a unique challenge, requiring sharp clinical awareness and customized anticoagulation strategies like fondaparinux or DOACs. This multifaceted case emphasizes the importance of adapting treatments to the complexity of thrombotic events. It also calls for deeper research to refine therapeutic approaches that ensure better patient outcomes while reducing long-term complications. Ultimately, this case underscores the critical need for innovation in managing rare, intersecting conditions.

## Informed consent

Informed consent was obtained from the patient for the publication of this case report and associated images. Institutional approval was secured from the Institutional Review Board (IRB) of the Medical Research Center of Hamad Medical Corporation (MRC-04-24-551).

## Conflicts of interest

None.
